# Dr. Adams, on Quackery

**Published:** 1806-04

**Authors:** Joseph Adams

**Affiliations:** Berner's Street


### *To the Editors of the Medical and Physical Journal.*

Gentlemen,

OUR countrymen are accused of being greater dupes to quackery than other nations. This imputation is I believe, unfounded, and arises more from the wealth of the kingdom increasing the facility with which quack medicines are advertized, and the numbers of people of all descriptions who are in a situation to encourage such impositions. Since the French revolution, it is well known that periodical publications of all kinds are much increased, and in a few years we shall probably find as large a portion of their columns engrossed by infallible remedies as meet our eyes every day in our own. In one instance they have stepped beyond us, for I do not recollect that any publication, calling itself a Review, has been used as the means of circulating the celebrity of such remedies, excepting on their wrappers, covers, or a few leaves tacked at the beginning and end. In a work, entitled La Revue ou Décade Philosophique, &c. lately instituted at Paris, is a paper too long to transcribe, but of which I shall offer enough to show that the whole is of such a nature, as would not easily find admission into any publication of the same kind in England. The paper is entitled, "*Observations sur un moyen nouveau de guérir l'Epilepsie, par M. Alphonse Leroy, Ancien Docteur Regent de la Faculte et Professeur de Medicine a Paris.*" The author begins by telling us, how much his labours had been directed to preserving children from a malady very common in the North of Europe. He next remarks, that in Russia, Denmark, and Sweden, *millions* of children become epileptic from the breast. That this probably arises from the habit of gin drinking among the nurses; ardent spirits made of grain being more deleterious than brandy; and the want of perspiration in the inhabitants of the North concurring to retain in the economy that gaseous principle which is the cause of this terrible disease. After this he enters into a deep disquisition on the causes which render spirits distilled from grain so much more deleterious than any other; and then assures us, that he has found epilepsy in grown people the effect of an old venereal taint, or of the itch.

To relieve so general and distressing a malady, M. Leroy informs us, that he has left no means untried not only in his own most laborious investigation, but, laying aside all prejudices, even in his enquiries after empirical remedies. He has also consulted many of his colleagues, among the rest M. Sabatier, the Nestor of French surgery; who acquainted him with a paper published by the French Academy in the year 1724. This paper mentions a green stone used by the savages of Oronoque, the virtues of which were very astonishing in the falling sickness. The extract given by M. Leroy is admissible for the period in which it was published, but that gentleman could have only one reason for bringing it forward in these times.

Les sauvages apportent quelquefois de la terre ferme ou de la rivierre de l'Oronoque une pierre verte qui est un remede etonnant contre le mal caduc. Il n'en faut que la grosseur d'une tête d'epingle; mais il y a deux maniéres de s'en servir; ou la porte dans une bague percée en dessous, de sorte que la pierre touche la chair; cela suffit; ou bien, on la fait entrer par une legere incision entre cuir et chair. Dans quelque partié du corps que ce soit, elle exerce toujours sa virtue. M. Hauterive a vu l'experience de la pierre appliquée de cette seconde maniere à une personne sujette sujette au mal caduc et qui depuis quinze ans n'a en aucune attaque. M. d'Hauterive en a un petit morceau en une bague, qu'il garde pour en secourir quelqu'un dans l'occasion.

Full of this, and some other observations, M. Leroy tells us, he has been examining all the green stones which re found in the rivers of the western world. He has also made enquiries in every possible way, and thinks he has procured green stones enough from Oronoque to make several experiments, and concludes by telling us of an extraordinary instance in which he cured a most deplorable case. It is unnecessary to give all the particulars; but the author conceives the efficacy of the remedy will be greatly increased by the incision, as his patient tried it ineffectually for some time, applied to different parts of the body without breaking the skin. Lest we should suppose that the stone applied in this manner, produced no other effect than a common issue, we are informed that the patient found no relief from one which had been kept open for some time.

M. Leroy concludes by some sage remarks on the importance of the part to which this green stone is applied, and anticipates many objections which he is aware will be made to his remedy.

On the whole, Gentlemen, I think you will agree with me, that this advertisement is not more ridiculous than the anodyne necklaces, the virtues of which, are I believe, beginning to wear out. What success M. Leroy may meet with in France, I pretend not to determine, but the proposal seems to me too absurd for this country, so celebrated for its credulity.

I remain, &c.

*Berner's Street, March* 3, 1806. JOSEPH ADAMS.

